# Detection Method for Three-Phase Electricity Theft Based on Multi-Dimensional Feature Extraction

**DOI:** 10.3390/s24186057

**Published:** 2024-09-19

**Authors:** Wei Bai, Lan Xiong, Yubei Liao, Zhengyang Tan, Jingang Wang, Zhanlong Zhang

**Affiliations:** 1 College of Electrical Engineering, Chongqing University, Chongqing 400044, China; 20173429@cqu.edu.cn (W.B.);; 2 Cincinnati Joint Co-Op Institute, Chongqing University, Chongqing 400044, China; 20211129@stu.cqu.edu.cn (Y.L.); 20211060@stu.cqu.edu.cn (Z.T.)

**Keywords:** electricity theft, data mining, Catch22-Conv-Transformer, three-phase system

## Abstract

The advent of smart grids has facilitated data-driven methods for detecting electricity theft, with a preponderance of research efforts focused on user electricity consumption data. The multi-dimensional power state data captured by Advanced Metering Infrastructure (AMI) encompasses rich information, the exploration of which, in relation to electricity usage behaviors, holds immense potential for enhancing the efficiency of theft detection. In light of this, we propose the Catch22-Conv-Transformer method, a multi-dimensional feature extraction-based approach tailored for the detection of anomalous electricity usage patterns. This methodology leverages both the Catch22 feature set and complementary features to extract sequential features, subsequently employing convolutional networks and the Transformer architecture to discern various types of theft behaviors. Our evaluation, utilizing a three-phase power state and daily electricity usage data provided by the State Grid Corporation of China, demonstrates the efficacy of our approach in accurately identifying theft modalities, including evasion, tampering, and data manipulation.

## 1. Introduction

Electricity, a cornerstone influencing industrial, technological, and economic development, experiences a steadily growing global demand [1]. As wind, hydropower, and nuclear power plants are increasingly deployed, power generation facilities now produce sufficient electricity to meet consumer needs while mitigating carbon emissions. However, technical losses (TL) and non-technical losses (NTL) within distribution systems hinder the complete transmission of electricity to end-users, resulting in substantial waste [2,3]. TL refer to the inherent losses incurred during transmission, transformation, distribution, and metering, which are necessary to maintain the operation of various power system components. Conversely, NTL primarily stem from abnormal electricity consumption behaviors, notably electricity theft at the distribution level, constituting controllable losses [4]. This global challenge has resulted in cumulative losses exceeding USD 96 billion annually [5]. In China, NTL account for 16% of total electricity generation, whereas in India, they surpass 25% [6]. Even in developed nations, these losses are non-negligible, with Canada and the United States incurring approximately USD 10 billion and USD 6 billion in annual economic losses, respectively [7].

In pursuit of the reliable, secure, economical, and efficient distribution and transmission of electricity, the latest power system known as the “Smart Grid (SG)” has been deployed in countries such as the United States and China [8]. The SG leverages advanced computing, networking, and measurement technologies, deploying numerous metering devices to collect electrical state data across various segments. These data enable power utilities to analyze consumer behavior with heightened accuracy [9,10]. Indeed, as the SG matures, the transition from traditional manual patrolling to automated data-driven detection of NTL has become feasible for power companies [11].

Currently, the detection of electricity theft is categorized primarily into three approaches based on the differences in detection technologies: hardware-based, data-driven, and game-theoretic methods [12,13]. Among these, game-theoretic research views electricity thieves and power utilities as rational competitors who devise strategies to maximize their gains while considering multiple risks [9,14]. However, due to its strong correlation with economics and psychology, this methodology deviates significantly from the approach presented in this paper and, thus, will not be directly compared.

Hardware-based approaches to electricity theft detection utilize sensors or specialized metering devices to monitor and detect energy utilization within power distribution lines with heightened precision [15,16]. Henriques developed an automated current measurement device that determines the presence of theft by comparing consumer energy consumption data against those of the main station [17]. Leite employed a combination of AMI, Phasor Measurement Units (PMUs), Intelligent Electronic Devices (IEDs), and Geographic Information Systems (GIS) to detect NTL [18]. Additionally, Zhou achieved high-precision detection of electricity theft with an accuracy rate of 97.6% by deploying Data Protection Relays (DPRs) at data protection centers and calculating anomaly rates using the Minimum Covariance Determinant method for each consumer [19]. Notwithstanding their precision, the high costs associated with equipment, frequent maintenance requirements, and the limitations in adapting to novel theft techniques significantly constrain the applicability of these hardware-based methods.

The data-driven approaches to detecting NTL can be broadly categorized into those based on power network state analysis and those based on machine learning. The former involves modeling the electricity consumption areas, analyzing sensor measurements and electrical parameters within the distribution network, and calculating network-wide parameters to identify NTL [20,21]. Kim introduced an intermediary monitoring instrumentation model based on the concept of unit network partitioning, constructing a system of linear equations through the analysis of power flow and energy balance, thereby facilitating effective detection of electricity theft [3]. These methods offer economic and efficiency advantages in accurately detecting theft within specific consumption areas. Nonetheless, their dependency on reliable data and detailed network topology structures renders them challenging to model, with limited portability across diverse consumption zones, thereby hindering their direct applicability in other areas [22].

Machine learning-based approaches excel at uncovering unknown yet valuable insights into user behavior, thereby facilitating the identification of electricity theft by utilities [23]. Table 1 encapsulates the datasets employed by researchers in this domain [24,25,26,27,28,29,30]. Among these, the State Grid Corporation of China (SGCC) dataset stands as the most prevalent for theft detection studies. Javaid devised a theft detection model utilizing the SGCC dataset, which extracts features through an attention-driven feature extractor and classifies potential thieves using an Echo State Network [24]. The Commission for Energy Regulation (CER) dataset, sourced from smart metering trials in Ireland, was leveraged by Li to develop a hybrid Convolutional Neural Network–Random Forest (CNN-RF) model, incorporating a clustering approach to detect consumer theft [28]. Furthermore, Zidi constructed an energy consumption dataset based on data provided by the United States Department of Energy and evaluated various models’ performance in detecting theft, offering utilities guidance in selecting more effective detection methods [30].

**Figure 1:**
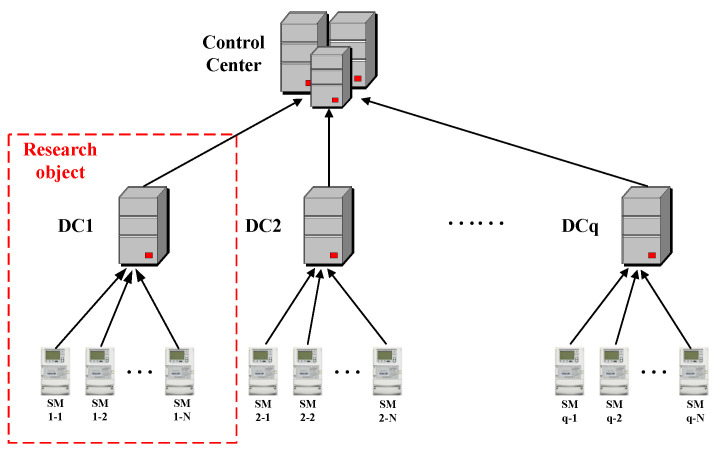
Composition and architecture of AMI.

These methodologies have been empirically validated on electricity consumption datasets, yielding promising results. However, their focus remains limited to a singular metric—daily or hourly energy usage—overlooking the complexity of the smart grid (SG) ecosystem. In reality, the SG, as a three-phase system, encompasses intricate electrical status information such as phase voltages, currents, and power factors, among others [31,32]. Notably, while evaluating model efficacy, prior works have predominantly emphasized accuracy metrics, neglecting the consequences of false positives. Excessive false positives can elicit customer dissatisfaction and complaints, necessitating substantial resources for verification by utility companies. To mitigate this, it is imperative to integrate a comprehensive analysis of diverse data types captured by AMI in the smart grid. AMI, comprising smart meters (SMs), Data Concentrators (DCs), and control centers, as illustrated in Figure 1, facilitates data transmission from SMs to DCs and subsequently to the control center, enabling comprehensive monitoring of energy transmission and consumption [33]. Additionally, metering facilities at DC installations measure total regional energy consumption.

Previous methodologies exhibit several limitations: some overlook the diverse nature of SG data, relying solely on single-dimensional energy consumption metrics to interpret user behavior, while others heavily rely on detailed electrical network topology information, significantly hampering their universality and scalability across different electricity supply regions. Consequently, a critical research challenge lies in mining the multifaceted electrical state data within the smart grid to facilitate precise and efficient electricity theft detection, while also ensuring the method’s general applicability and widespread adoption.

To address these limitations, our study concentrates on three-phase electricity usage within a designated area. Through simulation, we obtain authentic SG data and introduce a multi-dimensional feature extraction and Transformer-based electricity theft detection approach, namely the Catch22-Conv-Transformer model. This model harnesses the Catch22 feature set alongside three supplementary features to extract multi-dimensional characteristics from the diverse power consumption data collected by SMs and DCs. Subsequently, a convolutional network refines these features, which are then analyzed by a Transformer network equipped with a self-attention mechanism to identify various types of electricity theft. Our method’s effectiveness is validated across multiple datasets. The key contributions of this work are as follows:

- Addressing data imbalance through physically grounded simulations: To mitigate the ubiquitous challenge of data imbalance in electricity theft detection, we devised a solution rooted in physical principles. This involves the simulation of three-phase electricity consumption scenarios, leveraging both real-world three-phase SM data and the Sumlink environment. By recreating authentic power usage scenarios, we effectively address the issue of data imbalance that commonly plagues such studies.
- Multi-dimensional feature extraction and processing methodology: To enhance feature representation, our study introduces the integration of the Catch22 feature set alongside three supplementary features, specifically tailored for capturing diverse power consumption patterns. These features are then subjected to a convolutional network for refinement, ultimately facilitating more accurate electricity theft detection.
- Rigorous evaluation framework: To comprehensively evaluate the proposed approach, we employed a multifaceted assessment metric system. Furthermore, we validated the Catch22-Conv-Transformer model using two distinct datasets, each characterized by different data dimensionalities. This approach not only underscores the model’s adaptability across varied scenarios but also reinforces its robustness and reliability.

## 2. Materials and Threat Models

The dataset utilized in this research consists of three-phase power state data, collected by smart meters installed by the State Grid Corporation of China (SGCC), as depicted in Table 2. This comprehensive dataset captures the electrical profiles of 2118 users across a specified region, recorded at 15 min intervals from March to April 2023, yielding 96 daily measurements. The dataset includes A, B, and C phase voltages, currents, power factors, and periodic power fluctuations. Table 1 summarizes the basic data types and their abbreviations, where ‘M’ indicates the respective A, B, and C phases, ‘*t*’ represents the specific day, and ‘*d*’ denotes the sampling point within that day. Due to the difficulties in obtaining three-phase electricity theft data and their rarity, it is crucial to develop strategies to simulate electricity theft behaviors, thereby enriching the dataset with adequate samples. Before simulation, we assume that all users are honest and do not engage in electricity theft.

### 2.1. Data Pre-Processing

AMI is susceptible to external factors during operation, often resulting in incomplete and inconsistent data. Consequently, prior to simulating electricity theft behaviors, it is imperative to perform interpolation of missing data points. Additionally, following the acquisition of simulated data, normalization processing is necessary to ensure consistency.

#### 2.1.1. Missing Data Interpolation

Due to regular maintenance, occasional failures, and data loss of SMs during transmission, there may be missing data in the electric state data. The Lagrange interpolation method is used for data filling, as shown in Equation (1) [34].

(1) $$\begin{array}{l} L_{n}(x)=\sum_{i=-n}^{n}l_{i}(x)y_{i}\\ l_{i}(x)=\prod_{i=-n,i\neq j}^{n}\frac{x_{0}-x_{j}}{x_{i}-x_{j}} \end{array}$$

where *L_n_*_(*x*)_ represents the interpolation result, *l_i_*_(*x*)_ is the basis function corresponding to the Lagrange interpolation, *x*_0_ is the subscript number of the missing value, *x_i_* is the subscript number corresponding to the non-missing value *y_i_*. When interpolating missing values in a sequence, we extract 10 non-missing values before and after each missing value for the calculation. In case of consecutive missing values in a certain segment of the sequence, we search forward or backward in cycles of 96 points (1 day) to find non-missing values as the basis for calculation.

#### 2.1.2. Data Normalization

It is crucial to acknowledge that, even among SM data collected within the same electrical region, substantial numerical variations in current and voltage values may occur at distinct time points. To enhance computational efficiency and precision, normalization of these data, as detailed in Equation (2), is imperative.

(2) $$x^{\prime }=\frac{x-x_{\min}}{x_{\max}-x_{\min}}$$

where *x* signifies the value at a particular point within a specified sequence of power state data for an individual user. $x^{\prime }$ represents the corresponding normalized value of *x*. $x_{\max}$ denotes the maximum value encountered within that particular sequence, while $x_{\min}$ indicates the minimum value within the same sequence.

### 2.2. Traditional Electricity Theft Attack Model

In current research on data-driven electricity theft detection, researchers widely adopt six types of electricity theft attack models, as shown in the Table 3, to attack original electricity consumption data and obtain data from electricity theft users [35,36]. Here, $x_{t}^{d}$ represents the electricity consumption at time t on the *t*-th day, α represents the proportion of electricity theft, and mean( ) is the averaging function. Types 1 and 2 reduce electricity consumption by a certain proportion, while Type 3 subtracts a constant fixed value from electricity consumption. Types 4 and 5 set the user electricity consumption to zero and the recent average value, respectively. Type 6 reverses the order of electricity consumption to simulate behavior of consistently using electricity during low-price periods. Although these electricity theft attack models can increase the number of electricity theft samples to some extent, their interpretability is not obvious, making it difficult to represent complex electricity usage situations in three-phase electrical regions.

To address the issues in the above models, we propose a data generation method based on real datasets and simulations to mimic the electricity usage behavior of multiple users in three-phase electrical regions. The electricity theft behaviors we primarily focus on fall into three categories, each corresponding to real-world actions, as shown in Table 4.

**Figure 2:**
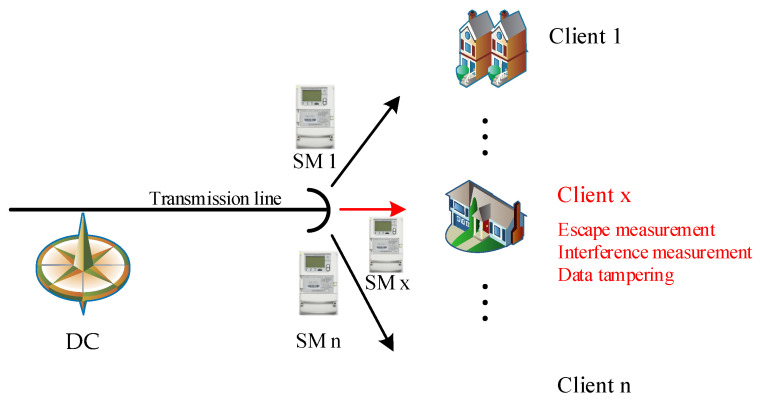
A schematic diagram of a three-phase electrical region.

We constructed a schematic diagram of a three-phase electrical region as shown in Figure 2, and randomly selected single or multiple users from the real dataset to simulate the electricity theft behaviors listed in Table 3. In the figure, DC denotes the Data Concentrator for the region, while SM represents the smart meters of individual users within the area. For ease of analysis and data processing, all users in the region were assigned unique identifiers ranging from 1 to n.This allowed us to obtain power state data from three-phase electricity theft users under the most realistic scenarios.

The impedance $R_{Mt}^{d}$ and reactance $X_{Mt}^{d}$ of user loads in each time period in Figure 2 are calculated using Equation (3).

(3) $$\begin{array}{l} R_{Mt}^{d}{=(U}_{Mt}^{d}\times \cos\varphi_{Mt}^{d}{)/I}_{Mt}^{d}\\ X_{Mt}^{d}{=(U}_{Mt}^{d}\times \sqrt{1-(\cos\varphi_{Mt}^{d}{)}^{2}}{)/I}_{Mt}^{d} \end{array}$$

where the voltage $U_{Mt}^{d}$, current $I_{Mt}^{d}$ and power factor $\cos\varphi_{Mt}^{d}$ of each phase of the user are given by the dataset described in Table 2.

The following methodologies were employed to simulate various electricity theft scenarios:

Evasion of Measurement:

- The measurement loop preceding the meter was short-circuited to mimic the bypassing of the meter.
- The phase sequence of the voltage and current measurement nodes on the meter were scrambled and reconnected to simulate phase shifting.

Interference with Measurement:

- Three constant-valued resistors were inserted into the meter’s measurement loop to emulate voltage and current diversion theft.
- Randomly varying resistors and capacitors were introduced into the measurement loop, mirroring electromagnetic interference theft.

Data Tampering:

- The impedance and reactance of a virtual circuit established upstream of SMs were configured to match the average values of the actual circuit, effectively replicating average value tampering.
- For the purpose of simulating the reversal of electricity usage timelines, the impedance and reactance of the virtual circuit were set to specific values in reverse chronological order.

### 2.3. Simulation Result

**Figure 3:**
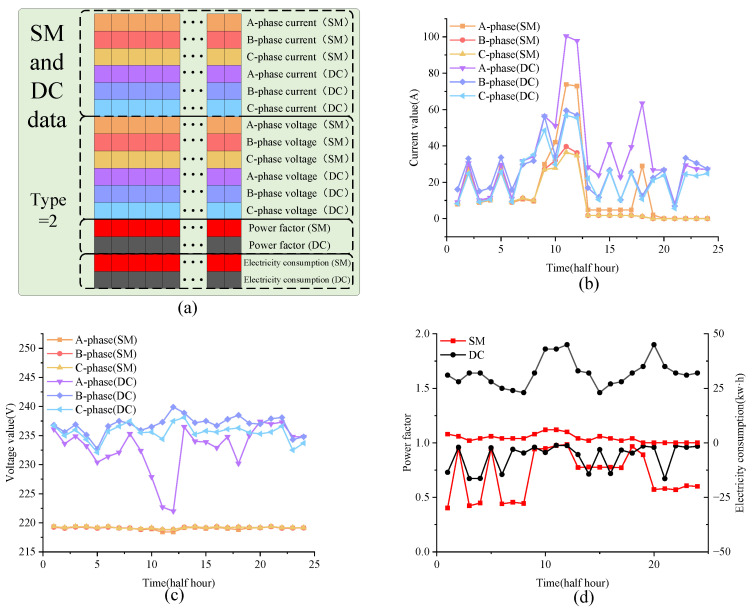
Simulation results for an electricity theft user (**a**–**d**). (**a**) Description of simulated data types. (**b**) Comparative analysis of three-phase current between DC and SM. (**c**) Comparative analysis of three-phase voltage between DC and SM. (**d**) Comparative analysis of power and power factor between DC and SM.

The simulation results for evading measurement electricity theft are presented in Figure 3. Specifically, Figure 3a showcases the diverse sequences of power state data pertaining to a particular user. These data encompasses instantaneous voltage and current readings for three phases, power factor, and periodic power fluctuations, all recorded by the SMs and DC in the designated area at 15 min intervals. The data are organized into an array with dimensions of 24 × 4 × 60 × 16, resulting in a matrix of 5760 × 16. Concurrently, the electricity theft committed by the user is categorized using labels: Type = 0 for honest users, Type = 1 for those evading measurement, Type = 2 for interfering with measurement, and Type = 3 for data tampering.

According to Figure 3b, the current trends measured by the user’s SM and DC align for an initial period but diverge sharply, with the DC-measured current experiencing a rapid decline thereafter. Conversely, Figure 3c reveals that while the DC-measured voltage exhibits fluctuations, the phase voltages captured by the SM remain stable at approximately 220 V. Furthermore, as observed in Figure 3d, the DC-measured power factor maintains a stable value of 0.9 with minor downward deviations, in contrast to the user’s SM, which registers abnormal fluctuations within the range of 0.9 to 0.5. The periodic power variation recorded by the SM also deviates significantly from the DC measurements. Consequently, within a three-phase power consumption area, the aberrant characteristics of electricity theft can be discerned from the multifaceted power state data collected by SMs and DCs, thereby enhancing the precision in identifying such behaviors.

## 3. Proposed Framework

**Figure 4:**
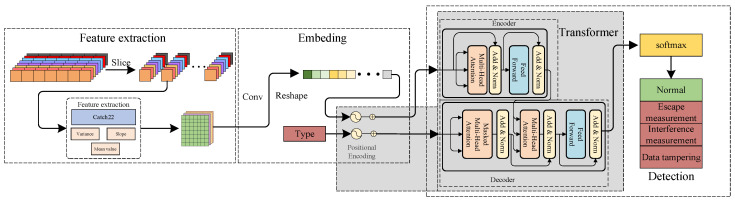
Overall framework of the model.

As shown in Figure 4, the Catch22-Conv-Transformer model mainly consists of three modules, and the electricity theft detection process is summarized as follows:

- Feature Extraction Module: The user data samples are segmented into sub-samples of 672 × 16 each. Catch22 and three supplementary feature extraction methods are used to extract multi-dimensional feature quantities, resulting in a feature set of 25 × 8 × 16.
- Embedding Module: Based on Conv, the feature set is transformed into a one-dimensional token sequence. The sequence and its corresponding category are encoded and then fed into the Transformer.
- Detection Module: Utilizing the encoder and decoder of the Transformer, the multi-head attention mechanism is employed in a higher-dimensional subspace to obtain attention distributions in different spaces in parallel. This captures the relationships between various feature values and categories, resulting in a probability distribution of the sequence across all categories. The softmax function is then applied to determine the category of the user.

### 3.1. Feature Extraction (Catch22)

The size of the three-phase user power state data is 5760 × 16, exhibiting the characteristics of “massive” and “high-dimensional” big data, and also possessing a temporal correlation. It is challenging to directly mine the objective laws and inherent relationships in these sequence data using traditional methods. Therefore, feature extraction is necessary to provide data support for monitoring electricity theft.

Catch22 (22 Canonical Time Series Characteristics) is a feature set consisting of 22 typical time series characteristics [37], derived from the 7658 time series features in the hctsa toolbox. During the screening process, hctsa removed 766 features that were sensitive to variance and mean. Subsequently, through validation conducted on more than 80 datasets, 2101 features with abnormal outputs were excluded. Hierarchical cross-validation was performed on the remaining features using a decision tree classifier, and the most important 22 features were selected based on balanced accuracy. The Catch22 feature set includes numerical distribution characteristics, linear and nonlinear autocorrelation, predictability, and fluctuation scales of time series.

**Figure 5:**
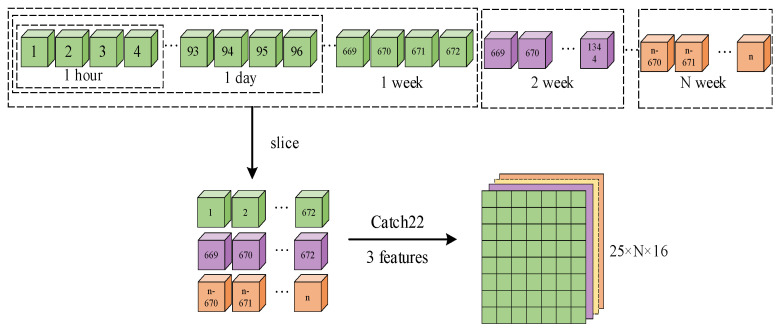
Feature extraction process.

The local characteristics of time series must be monitored. Therefore, subsequences with a length of 24 × 4 × 7 (one week) are selected each time for feature extraction, and three additional feature types are incorporated. The mean can distinguish subsequences with similar shapes but different amplitudes, the variance can reflect the dispersion of subsequence values, and the slope can indicate the trend of the subsequence. The feature extraction process of this study is illustrated in Figure 5, where a total of 25 features are extracted for user time subsequences, namely 22 Catch22 features in addition to mean, variance, and slope.

### 3.2. Embedding and Detection (Conv-Transformer)

Transformer is a deep learning model based on the self-attention mechanism, which processes all input data synchronously at each time step. It captures long-term dependencies and local features in the data while improving the overall efficiency of the network.

**Figure 6:**
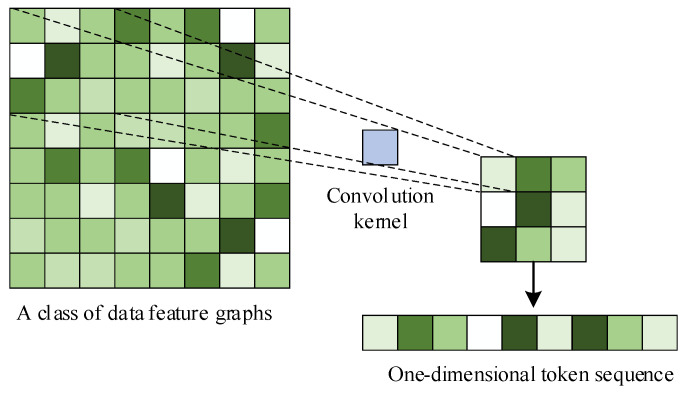
Convolution reshape.

During data processing, convolutional operations are performed on the input features, followed by slicing and flattening of the feature maps obtained from the convolution layers. This transforms the feature data into a one-dimensional token sequence required by the Transformer, as illustrated in Figure 6.

**Figure 7:**
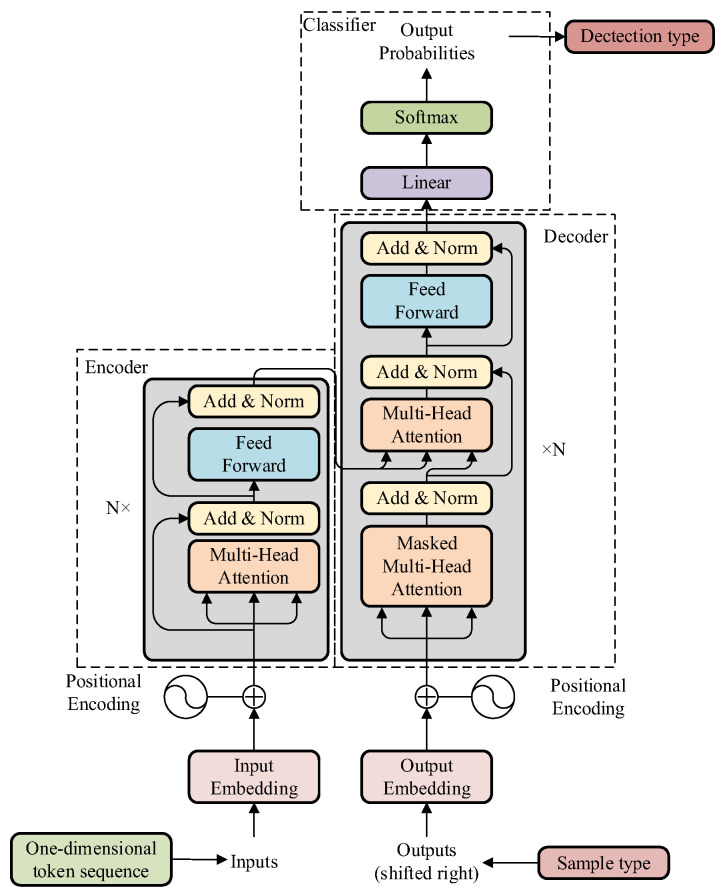
Overall architecture of Transformer.

The structure of the Transformer is illustrated in Figure 7, with its overall architecture divided into three modules: the embedding module, the encoder–decoder module, and the classification module [38]. In addition, residual join and layer normalization (Add & Norm) work together in each module of the model to improve the training efficiency and performance of the model.

#### 3.2.1. Embedding

The embedding module transforms the input sequence data into vectors of the same length but higher dimensions. To leverage the temporal and spatial information of the data, positional encoding is added to the output vectors. The positional encoding has the same dimension as the input embedding, and its encoding is represented by Equation (4).

(4) $$\begin{array}{l} F_{\mathrm{PE}}{(F}_{\mathrm{pos}},2j{)=\sin(F}_{\mathrm{pos}}{/10000}^{\frac{2j}{d_{\mathrm{model}}}})\\ F_{\mathrm{PE}}{(F}_{\mathrm{pos}},2j{+1)=\cos(F}_{\mathrm{pos}}{/10000}^{\frac{2j+1}{d_{\mathrm{model}}}}) \end{array}$$

where $F_{\mathrm{pos}}$ denotes the position of each input data in the sequence, *j* represents the dimension of the positional encoding, and $d_{\mathrm{model}}$ is the set transformation dimension.

#### 3.2.2. Encoder–Decoder

The encoder and decoder modules of Transformer are stacked with multiple encoders and decoders that share the same structure. As shown in Figure 7, an encoder consists of two sublayers: the first is the multi-head attention mechanism, and the second is a fully connected feedforward neural network. Residual connections are employed around each sublayer, and layer normalization is used to accelerate model convergence and mitigate the issue of overfitting. The decoder has a similar structure to the encoder, with an additional sublayer inserted that applies a masked multi-head attention mechanism to the output embeddings. By performing addition and shifting operations on these output embeddings, it ensures that when generating the output at the current position, the decoder can only rely on the known outputs at positions less than the current one.

**Figure 8:**
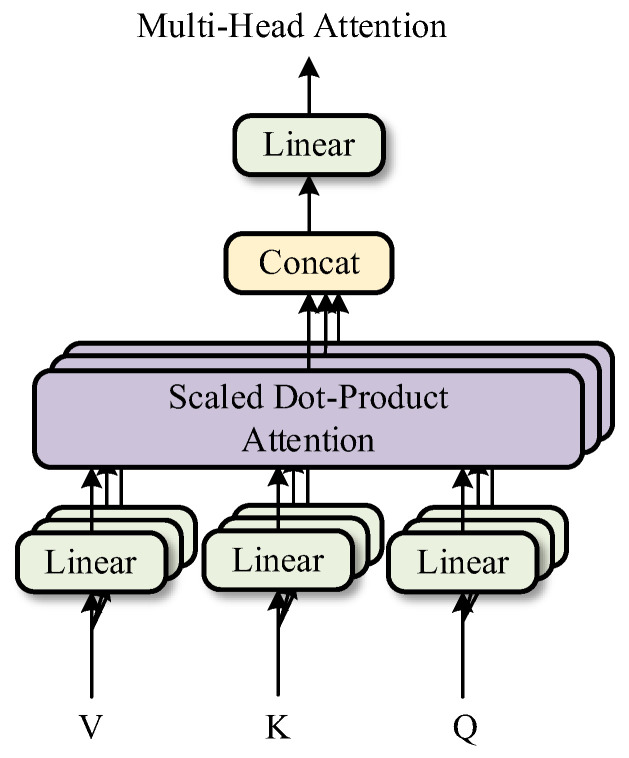
The multi-head attention mechanism.

The multi-head attention mechanism is the core of Transformer, consisting of a self-attention layer, a concatenation layer, and a linear transformation layer, as illustrated in Figure 8. By integrating multiple parameter-independent self-attention networks, it excavates the dependencies of data from different perspectives, representing the temporal and spatial relationships of data more accurately than traditional attention mechanisms. Taking the *i*-th self-attention network as an example, the calculation method of the attention matrix for each network is explained. Firstly, three different linear projection matrices $Q_{i}$, $K_{i}$ and $V_{i}$, are initialized to map the input feature sample X to the query, key, and value matrices, respectively. The calculation process of each parameter is shown in Equation (5).

(5) $$\left\{\begin{array}{l} Q_{i}{=\mathrm{XW}}_{i}^{Q}\\ K_{i}{=\mathrm{XW}}_{i}^{K}\\ V_{i}{=\mathrm{XW}}_{i}^{V}\\ H_{i}=\mathrm{softmax}(\frac{Q_{i}K_{i}^{T}}{\sqrt{d}}{)V}_{i} \end{array}\right.$$

where $H_{i}$ represents the output result of the *i*-th self-attention network, *d* denotes the dimension of the linear projection matrix, softmax( ) is the activation function.

Then, by concatenating the data results from multiple self-attention networks, the final output attention value matrix is obtained, which helps the model to focus more on the important parts of the input, as shown in Equation (6).

(6) $${H=\mathrm{Concat}(H}_{1}{,H}_{2},\cdots {,H}_{n}{)W}^{0}$$

where Concat( ) represents the concatenation function, $W^{0}$ denotes the weight function.

#### 3.2.3. Classification

The data processed by the encoder–decoder is mapped to an appropriate dimension through a linear layer output. Then, the softmax function is used to convert the output into probability values between 0 and 1, and the category with the highest probability is obtained as the classification result.

### 3.3. Loss Function

The proposed model is trained using both the Cross-Entropy (CE) and Dice loss functions. The CE loss function is a representative function in classification tasks, as shown in Equation (7).

(7) $$L_{CE}=-\frac{1}{n}\sum_{i=1}^{n}y_{i}\log(p_{i})$$

where *y_i_* and *p_i_* represent the labels and model predictions, respectively, and *n* denotes the number of categories.

In fact, electricity theft users constitute a minority of samples. The Dice loss function has significant advantages in handling such class imbalances, as it considers not only the current feature predictions but also the neighboring features. Its function is shown in Equation (8).

(8) $$L_{Dice}=1-\frac{1}{n}\sum_{i=1}^{n}\frac{2\sum_{j=1}^{H}\sum_{k=1}^{W}P_{ijk}y_{ijk}}{\sum_{j=1}^{H}\sum_{k=1}^{W}p_{ijk}^{2}+\sum_{j=1}^{H}\sum_{k=1}^{W}y_{ijk}^{2}+\varepsilon }$$

where *H* and *W* represent the height and width extracted from the features, takes the value of 1 × 10^−5^ to avoid a zero denominator.

The CE and Dice loss functions are incorporated into the model, and the loss function is shown in Equation (9).

(9) $$L_{t}=\alpha \times L_{ce}+(1-\alpha )\times L_{Dice}$$

## 4. Results

This study constructed an electricity theft detection dataset for three-phase power state areas using real SM data and conducted extensive experiments to evaluate the effectiveness of our proposed Catch22-conv-Transformer model. The model was built on PyTorch and run on hardware equipment consisting of a 13th Gen Intel Core i5-13400F 2.5 GHz CPU and a GTX 4060Ti 12G GPU. The Adam optimizer was used for training, with the initial learning rate set to 0.01 and adjusted using a cosine annealing learning rate schedule.

### 4.1. Dataset

In the experiment, two datasets, as shown in Table 5, were used to test the performance of the proposed model. Dataset 1 was provided by the State Grid of China and included three-phase electricity consumption data from users as well as power state data from electricity theft users generated by Simulink. This dataset comprised three-phase power consumption data from 6486 users’ SM and DC, ranging from March 2023 to April 2023, with 96 measurement points per day. Among these users, there were 3243 normal users and 1081 users of each of the three types of electricity theft. Dataset 2 was released by the State Grid of China and contained daily electricity consumption data from a total of 43,272 power users over 1035 days, from 1 January 2014 to 31 October 2016 [16]. Among these users, there were 38,757 normal users and 3615 electricity theft users.

### 4.2. Metrics

**Figure 9:**
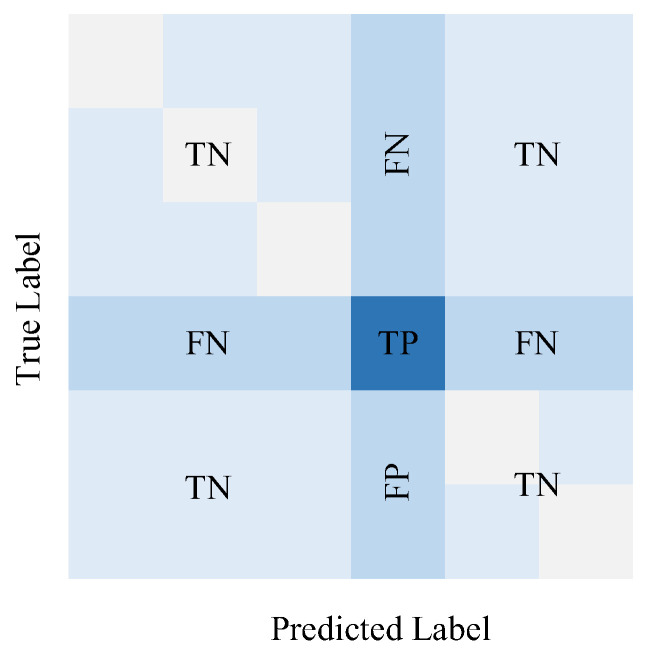
Confusion matrix for classification.

The confusion matrix, as shown in Figure 9, is used to compare the classification results with the actual values, providing a visual representation of the classification status for each category. In this matrix, the rows represent the true categories, while the columns represent the predicted categories.

For a specific category, TP (true positive) indicates that users of this category are correctly predicted; FP (false positive) indicates that users not belonging to this category are predicted as belonging to this category, TN (true negative) indicates that users not belonging to this category are correctly predicted as not belonging to this category; and FN (false negative) indicates that users of this category are incorrectly predicted as not belonging to this category.

Evaluation metrics include ACC (Accuracy), FPR (False Positive Rate), Precision (PR-E), Recall, and F1. Specifically, ACC represents the proportion of correctly predicted samples among all samples; FPR indicates the ratio of actual non-target users who are predicted as target users among all samples, Precision (PRE) refers to the percentage of actual target users among all predicted target users, Recall measures the proportion of actual target users who are correctly predicted as target users among all actual target users, F1 is a comprehensive metric for evaluating the model. The calculations of these metrics are shown in Equation (10).

(10) $$\begin{array}{l} \mathrm{ACC}(\%)=(\mathrm{TP}+\mathrm{TN})/(\mathrm{TP}+\mathrm{TN}+\mathrm{FP}+\mathrm{FN})\times 100\\ \mathrm{FPR}(\%)=\mathrm{FP}/(\mathrm{TP}+\mathrm{FN})\times 100\\ \mathrm{PRE}(\%)=\mathrm{TP}/(\mathrm{TP}+\mathrm{FP})\times 100\\ \mathrm{Recall}(\%)=\mathrm{TP}/(\mathrm{TP}+\mathrm{FN})\times 100\\ F1(\%)=(2\mathrm{PRE}*\mathrm{Recall})/(\mathrm{PRE}+\mathrm{Recall})\times 100 \end{array}$$

Due to the relatively small proportion of electricity theft users, the Receiver Operating Characteristic (ROC) curve is adopted to evaluate the performance of the model. Different points on the curve represent the detection effectiveness of the model for varying proportions of electricity theft users and normal users. The vertical axis of the curve is Recall. A steeper curve indicates a larger area under the curve (AUC), which signifies better performance.

### 4.3. Experiments

The proposed model is primarily designed for detecting electricity theft in three-phase users. Therefore, experiments were initially conducted using Dataset 1. The dataset was mixed with electricity theft users and normal users, and then divided into training, testing, and validation sets in a ratio of 3:1:1.

**Figure 10:**
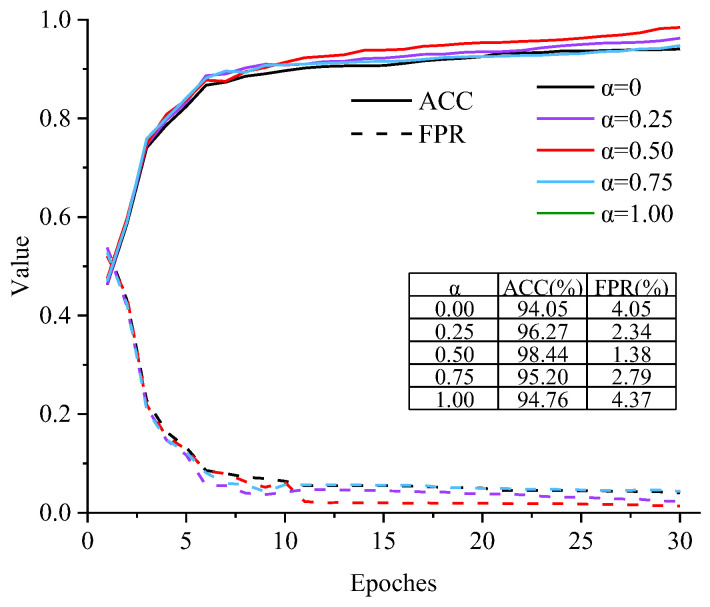
Performance curves of different loss function weights.

To determine the optimal value for the weight α in the loss function, we conducted experiments with α values set at 0.00, 0.25, 0.50, 0.75, and 1.00. The experimental results are shown in Figure 10. When α is equal to 0 or 1, it represents the use of the CE loss function and the Dice loss function, respectively. In these cases, the model’s ACC is 94.05% or 94.76%, respectively, and the FPR is 4.05% or 4.37%, respectively. When the CE loss and Dice loss functions jointly optimize the network parameters, the model’s ACC increases and FPR decreases, indicating that both loss functions have an impact on network performance. When α is 0.50, the model’s ACC reaches 98.44%, and the FPR is only 1.38%, suggesting that this weight ratio achieves the best network performance. This weight ratio is applied in subsequent experiments.

**Figure 11:**
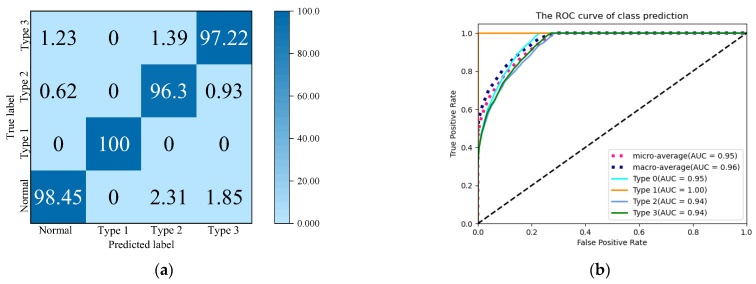
Experimental results. (**a**) Confusion matrix for each type of electricity theft; (**b**) ROC curves for the detection of each type of electricity theft.

To analyze the detection capabilities of the proposed model for the three major electricity theft groups: evasion of measurement, interference with measurement, and data tampering, we plotted the results as a confusion matrix shown in Figure 11a. In this matrix, the horizontal axis represents the predicted categories of the model, the vertical axis represents the true categories of the model, and the values in the matrix represent the proportion of users in each category that the model predicts correctly.

### 4.4. Construction of References

The recent works related to electricity theft detection have all used data from the daily electricity consumption dataset released by China’s State Grid, referred to as Dataset 2. The methods employed and their effects are shown in Table 6.

To demonstrate the effectiveness of the proposed model in detecting electricity theft users on one-dimensional sequential data, experiments were conducted using Dataset 2 and compared with the electricity theft detection models presented in Table 6.

**Figure 12:**
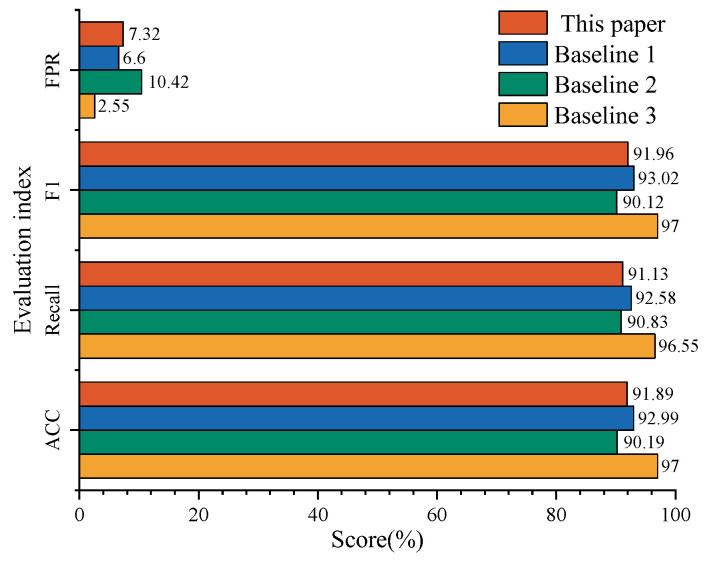
Comparison with other models.

In this study, an electricity theft attack model from Table 3 was used to generate 18,000 samples for electricity theft detection, serving as a supplement to address the issue of data imbalance. Subsequently, normal samples were mixed with electricity theft samples to construct the dataset, which was then divided into training, validation, and testing sets in a ratio of 4:1:1 for model training and validation. The evaluation results of the proposed method and the comparison methods are shown in Figure 12.

According to the data, the proposed model achieved an ACC of 97.00%, a Recall of 96.55, and an F1 of 97.00, which are all higher than those of the other comparison methods. Furthermore, the FPR of the proposed model is only 2.55%, significantly lower than that of the other methods, which indicates a lower false detection rate for electricity users, demonstrating the superior performance and higher accuracy of the proposed model.

## 5. Discussion

Practical Application Significance:

This study presents an innovative technique for detecting electricity theft in industrial manufacturing, commercial buildings, and agricultural sectors, where three-phase power regions are prevalent. This technique leverages four pivotal electrical state parameters—voltage, current, power factor, and energy consumption—collected by the AMI, effectively identifying theft activities. Notably, the approach eliminates the necessity for detailed network topology information, thereby significantly enhancing its universal applicability and scalability in real-world applications.

Potential Challenges:

By incorporating a broader spectrum of three-phase electrical state data, the proposed method substantially improves the accuracy of electricity theft detection. However, this augmentation is accompanied by a notable surge in data volume, inevitably leading to a decrease in computational speed for detection. As data sizes continue to escalate, optimizing the detection model for heightened efficiency becomes a pivotal challenge requiring immediate attention.

Limitations:

This research is grounded on the premise of relatively well-established smart grid deployments, a scenario prevalent in nations like China and the USA, where abundant and stable data provide a solid foundation. Nevertheless, it is imperative to acknowledge that for countries where power infrastructure is still evolving, the proposed method might encounter practical difficulties due to current constraints.

Future Research Directions:

In three-phase power systems, electrical parameters are intricately interdependent. Integrating these constraints into the electricity theft detection model as auxiliary analysis can markedly accelerate detection speeds. Looking ahead, deriving the inherent relationships between parameters promises to better equip us in addressing the mounting data challenges of tomorrow.

## 6. Conclusions

This paper proposes an electricity theft detection method for power state data measured by SM and DC in three-phase power consumption areas. To address the data imbalance issue in the detection process, a simulation approach is applied to combine Simulink software R2022 b with real three-phase data. The experimental results demonstrate that the proposed detection method can effectively handle multi-dimensional power consumption data and detect three types of electricity theft groups among three-phase users, such as evasion, interference, and data tampering, with ACC of 96.3%, 100%, and 98.45%, respectively. Furthermore, a comparison with three representative electricity theft detection methods on the SGCG dataset shows an ACC of 97% and an FPR of only 2.55%, highlighting the advantages of our method in terms of high detection accuracy and low false detection rate. The proposed method is capable of detecting electricity theft users in three-phase power consumption groups, and the model is expected to be deployed on the cloud servers of power companies to automatically detect electricity theft.

## Figures and Tables

**Table 1:** Overview of the dataset used in the field of energy theft.

Dataset	Time Stamp	Duration	Country	Data Type
SGCC [24,25,26]	1 day	January 2014–October 2016	China	Electricity consumption
CER [27,28,29]	30 min	January 2009–December 2010	Ireland	Electricity consumption
Electricity Theft [30]	1 h	Not mentioned	USA	Electricity consumption

**Table 2:** The primary data types and corresponding abbreviations.

Data Types	Corresponding Abbreviation
Phase Current	$I_{Mt}^{d}$
Phase Voltage	$U_{Mt}^{d}$
Phase Power Factor	$\cos\varphi_{Mt}^{d}$
Periodic Power Variation	$W_{t}^{d}$

**Table 3:** Electricity theft attack models.

Type	Formulation
1	$f_{2}(x_{t}^{d})=ax_{t}^{d},0.1<a<0.8$
2	$f_{2}(x_{t}^{d})=a_{t}^{d}x_{t}^{d},0.1<a<0.8$
3	$f_{3}(x_{t}^{d})=\max\{x_{t}^{d}-\gamma ,0\},\gamma <\max(x_{t}^{d})$
4	$f_{4}(x_{t}^{d})=0$
5	$f_{5}(x_{t}^{d})=mean\{x_{t=1,2,\cdots ,n}^{d}\}$
6	$f_{6}(x_{t}^{d})=x_{t=n,n-1,\cdots ,1}^{d}$

**Table 4:** Categories of electricity theft and corresponding behaviors.

Numbers	Category	Behavior
Type 1	Evasion	Bypassing the electricity meter by tampering with the connection
		Manipulating the phase shift between current and voltage inputs to the meter
Type 2	Interference	Introducing resistive shunt or voltage divider into the measurement circuit or
		employing electromagnetic interference to disrupt measurement accuracy
Type 3	Data Tampering	Substituting meter data with daily averages or
		reversing the transmission of daily electricity usage information from the meter

**Table 5:** Introduction of the datasets.

Dataset	Total Number of Samples	Data Type	Sample Category			
1	6486	Three-phase electricity state data for various types	Normal	Evasion	Interference	Data modification
			3243	1081	1081	1081
2	43,272	Daily electricity consumption	Normal	Electricity theft		
			38,757	3615		

**Table 6:** Comparative models.

Literature	Number	Method	ACC
[39]	Baseline 1	CatBoos	93.38%
[29]	Baseline 2	AEA-GRU-Feedforward t	95.80%
[40]	Baseline 3	RNN-BiLSTM-CRF	93.05%

## Data Availability

Data will be made available on request.
